# Establishing spectrochemical changes in the natural history of oesophageal adenocarcinoma from tissue Raman mapping analysis

**DOI:** 10.1007/s00216-020-02637-1

**Published:** 2020-04-25

**Authors:** Ishaan Maitra, Camilo L. M. Morais, Kássio M. G. Lima, Katherine M. Ashton, Danielle Bury, Ravindra S. Date, Francis L. Martin

**Affiliations:** 1 School of Pharmacy and Biomedical Sciences, University of Central Lancashire, Preston, PR1 2HE UK; 2 Institute of Chemistry, Biological Chemistry and Chemometrics, Federal University of Rio Grande do Norte, Natal, 59072-970 Brazil; 3 Lancashire Teaching Hospitals NHS Foundation Trust, Royal Preston Hospital, Preston, PR2 9HT UK; 4 Blackpool Teaching Hospitals NHS Foundation Trust, Blackpool Victoria Hospital, Blackpool, FY3 8NR UK

**Keywords:** Barrett’s oesophagus, Oesophageal adenocarcinoma, Principal component analysis, Raman spectroscopy, Raman mapping

## Abstract

Raman spectroscopy is a fast and sensitive technique able to identify molecular changes in biological specimens. Herein, we report on three cases where Raman microspectroscopy was used to distinguish normal vs. oesophageal adenocarcinoma (OAC) (case 1) and Barrett’s oesophagus vs. OAC (cases 2 and 3) in a non-destructive and highly accurate fashion. Normal and OAC tissues were discriminated using principal component analysis plus linear discriminant analysis (PCA-LDA) with 97% accuracy (94% sensitivity and 100% specificity) (case 1); Barrett’s oesophagus vs. OAC tissues were discriminated with accuracies ranging from 98 to 100% (97–100% sensitivity and 100% specificity). Spectral markers responsible for class differentiation were obtained through the difference-between-mean spectrum for each group and the PCA loadings, where C–O–C skeletal mode in β-glucose (900 cm^−1^), lipids (967 cm^−1^), phosphodioxy (1296 cm^−1^), deoxyribose (1456 cm^−1^) and collagen (1445, 1665 cm^−1^) were associated with normal and OAC tissue differences. Phenylalanine (1003 cm^−1^), proline/collagen (1066, 1445 cm^−1^), phospholipids (1130 cm^−1^), CH_2_ angular deformation (1295 cm^−1^), disaccharides (1462 cm^−1^) and proteins (amide I, 1672/5 cm^−1^) were associated with Barrett’s oesophagus and OAC tissue differences. These findings show the potential of using Raman microspectroscopy imaging for fast and accurate diagnoses of oesophageal pathologies and establishing subtle molecular changes predisposing to adenocarcinoma in a clinical setting.

**Electronic supplementary material:**

The online version of this article (10.1007/s00216-020-02637-1) contains supplementary material, which is available to authorized users.

**Graphical abstract:**
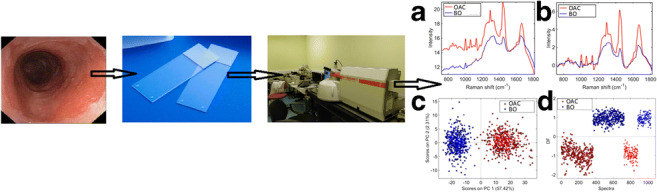
Graphical abstract demonstrating how oesophageal tissue is processed through Raman mapping analysis in order to detect spectral differences between stages of oesophageal transformation to adenocarcinoma

## Introduction

Oesophageal adenocarcinoma (OAC) is an aggressive disease which usually presents de novo and late with a poor prognosis. In the UK, the overall OAC 5-year survival rate is as low as 19% [1]. It is uncertain if alcohol and smoking contribute to the development of OAC. However, there is a proven association between adenocarcinoma and Barrett’s oesophagus, a condition that appears to arise in response to chronic inflammation from gastro-oesophageal reflux disease (GORD) [2, 3]. Other recognised risk factors for OAC include scleroderma, achalasia and Zollinger–Ellison syndrome [2]. The metaplasia–dysplasia–adenocarcinoma sequence is not only related to changes in ploidy and loss of heterozygosity, but genetic alterations in *P16* (CDKN2A) and *TP53* [4]. If dysplasia can initially be diagnosed accurately with adjuncts to histology, this would benefit earlier treatment and prevent the burden of patients developing OAC.

The application of Raman spectroscopy combined with chemometric approaches is a novel approach to delineate oesophageal diseases in human tissue. As an overall application, it has the potential to provide non-invasive, reagent-free and objective diagnosis of oesophageal dysplasia in vitro and aid histopathological diagnosis. To date, Raman spectroscopy has been applied to investigate diagnostic potential in the identification of oesophageal pre-malignant and malignant conditions. There are only a few studies using Raman spectroscopy looking at the identification of benign disorders of the oesophagus. An earlier diagnosis of dysplasia in benign oesophageal conditions such as Barrett’s oesophagus patients would ultimately enable the reduction of invasive and more expensive surgical options when disease requiring intervention has developed.

Herein, we report three cases of OAC where initial previous pathologies of gastro-oesophageal junction (GOJ) mucosa were normal squamous epithelium in one case and intestinal metaplasia being the initial pathology in the other two cases. The first case of OAC was diagnosed 3 months after an initially normal oesophagogastricduodenoscopy (OGD). The second was 2 years after their previous OGD for surveillance for Barrett’s oesophagus. The third was 2 ½ years after their previous OGD for surveillance for Barrett’s oesophagus. There is currently no clear recognition of biomarkers of the tissue spectrochemical changes that distinguish between the different stages of disease in an individual patient’s disease progression to oesophageal adenocarcinoma. Our aim is to understand and identify spectral differences using Raman spectroscopic mapping between both histological grades in these three illustrated cases.

### Case 1 (de novo OAC)

A 65-year-old lady presented to an Upper Gastrointestinal (GI) Clinic with a long-standing history of volume reflux. She had experienced dysphagia to solids and liquids over the past 2 months, with a sensation of food getting stuck at the level of her epigastrium. At the time, she denied any sinister features of malignancy such as weight loss or anaemia. Her past medical history included mild chronic obstructive pulmonary disease (COPD) and hypertension. She was a non-smoker and was tee-total. An urgent upper GI endoscopy revealed mild distal oesophagitis with normal squamous epithelium encountered on biopsy. Her *Campylobacter*-like organism (CLO) test was negative and she was subsequently discharged.

She presented 3 months later with a history of progressive dysphagia and a weight loss of 2 stone. Clinical examination was unremarkable and no sinister signs of pathology was seen on haematological and biochemical testing. An upper GI endoscopy however revealed a mid- to distal-oesophageal stricture suspicious of OAC. This was confirmed on biopsy. Further staging computerised tomography (CT) imaging revealed metastatic OAC (T4N2M1) with distal spread to her thoraco-lumbar spine and proximal femurs bilaterally. She had a metallic stent inserted under radiological guidance for symptomatic control. She declined further oncological input and unfortunately passed away 2 months since her malignant diagnosis.

### Case 2 (Barrett’s oesophagus to OAC)

A 69-year-old male with a background of Barrett’s oesophagus and lower limb peripheral vascular disease presented at the endoscopy department for his 2-year Barrett’s surveillance gastroscopy. He developed a short 1-month history of dysphagia to solids and a weight loss of 1 stone with reduced appetite prior to his surveillance gastroscopy. He was an ex-smoker (stopped 10 years ago; previously 15 pack years) and was tee-total. Clinical examination and prior haematological and biochemical tests were unremarkable. His previous OGD revealed uncomplicated junctional intestinal metaplasia consistent with Barrett’s oesophagus with no dysplasia. His most recent endoscopy identified a junctional OAC.

Staging CT imaging identified T3N2 disease with no evidence of distal metastatic disease. This was confirmed with staging laparoscopy performed a month after his initial endoscopic malignant diagnosis. A metallic stent was inserted under radiological guidance for symptomatic control and he has had 2 cycles of neoadjuvant chemotherapy with a view to cardio-oesophagectomy followed by adjuvant chemotherapy.

### Case 3 (BO to OAC)

**Fig. 1:**
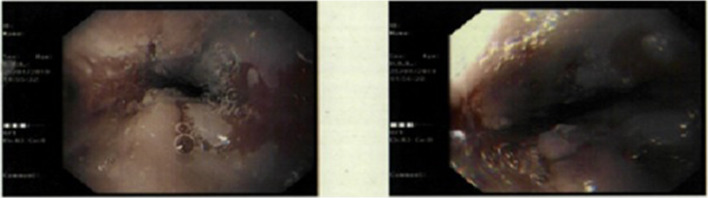
Ulcerative lesion on background of Barrett’s oesophagus (35 cm from incisors, 1 cm length)

A 75-year-old male with a background of Barrett’s oesophagus and type II diabetes mellitus with peripheral neuropathy presented at the endoscopy department for his 2-year Barrett’s oesophagus surveillance gastroscopy. His past surgical history included recurrent hiatus hernia repairs resulting in exertional dyspnoea. He had no new upper GI symptoms prior to his surveillance gastroscopy. He was an ex-smoker (stopped 20 years ago; previously 25 pack years) and was tee-total. Clinical examination and prior haematological and biochemical tests were unremarkable. His previous upper GI endoscopy revealed uncomplicated junctional intestinal metaplasia consistent with Barrett’s oesophagus with no dysplasia. His most recent endoscopy identified findings consistent with a junctional OAC (Fig. 1).

Staging CT imaging identified T3N1 disease with no evidence of distal metastatic disease. This was confirmed with staging laparoscopy performed 2 months after his initial endoscopic malignant diagnosis. The patient is currently undergoing his first cycles of neoadjuvant chemotherapy with a view to cardio-oesophagectomy followed by adjuvant chemotherapy.

## Materials and methods

### Ethical approval

Ethical approval was granted by the East of England - Cambridge Central Research Ethics Committee from 2015 (Archival gastro-intestinal tissue, blood, saliva and urine collection; REC reference: 18/EE/0069; IRAS project ID: 242639).

### Pre-sampling preparation

Archival oesophageal tissue samples were acquired from the histopathology laboratory at the Royal Preston Hospital from February to June 2019. Formalin-fixed tissue blocks are embedded in paraffin wax at room temperature at 20 °C as this ensures durability for long-term storage without deterioration of the sample architecture. Presently in the field of Raman spectroscopy, there is lack of consensus with regard to a standard protocol for de-paraffinisation of paraffin-embedded sections [5]. De-parrafinisation was hence performed prior to commencing Raman measurements using local hospital protocols employing three washes in fresh xylene and ethanol. Parallel sections of 10-μm thickness were prepared on FisherBrand™ slides (aluminium foil-covered; for spectroscopy) or of 4-μm thickness were prepared on FisherBrand™ slides (for histology). Parallel samples are used so that each section closely resembles the other sections, thus ensuring correlation between histology and spectroscopic measurements. An independent consultant histopathologist identified sections of the cut biopsies for an overall accurate representative analytical study of the tissue. This was to ensure that spectral measurements would be taken from the appropriate area and from the same area for the differing technologies to avoid heterogeneity in the cut tissue samples.

For each sample, the H&E section was scanned onto computer software. This allowed the regions that best reflect the overall diagnosis to be highlighted and labelled. Ten regions were selected for each sample and for each modality of analysis. Once prepared, the slides were transported in wooden slide boxes to the Biomedical Research Laboratory. All of the samples were stored in a de-humidified glass container to prevent condensation and physical damage.

### Raman mapping measurement

Raman spectra and mapping were collected with an InVia Renishaw Raman spectrometer coupled with a charge-coupled device (CCD) detector and a Leica microscope. A 200-mW laser diode was used at a wavelength of 785 nm with a grating of 1200 lines/mm. A silicon wafer was used to calibrate the Raman shift wavenumber value as it has a single sharp peak at 520.4 cm^−1^, which was used as the reference point. Streamline mapping was performed by moving the sample on the motorised stage under the laser beam. The size and area of the section to be mapped was based on the regions selected by the independent consultant histopathologist after high-resolution H&E stain analysis. On average, ten regions from each sample were mapped with a diverse range of size area depending on the size of the area of interest. The larger maps were typically from samples of adenocarcinoma, which had a single larger section of interest as compared to the other pathologies that had multiple smaller areas of interest. The measurements were made using a 785-nm laser (10% power, 30 mW) with × 50 zoom magnification. For each pixel in the Raman mapping image, a Raman spectrum in the range between 725 and 1813 cm^−1^ (1 cm^−1^ spectral resolution) was recorded.

### Data pre-processing and analysis

The data analysis was performed within MATLAB® R2014b (MathWorks, Inc., USA) using the Classification Toolbox for MATLAB [6], the HYPER-Tools toolbox for MATLAB [7], and in-house-developed algorithms. Firstly, the three-dimensional (3D) Raman mapping images were loaded into MATLAB and unfolded into two-dimensional (2D) structures containing *n* rows (number of spectra) and *m* columns (number of wavenumbers). Thereafter, each spectrum underwent pre-processing by Savitzky–Golay smoothing (21 points window, 2nd order polynomial fitting) and automatic weighted least squares (AWLS) baseline correction. Then, the resulting pre-processed spectra were split into training (70%) and validation (30%) sets using the Kennard–Stone algorithm [8], and then they were used for exploratory analysis via principal component analysis (PCA) and classification through principal component analysis linear discriminant analysis (PCA-LDA). The training set was used for model construction and optimisation, while the validation set for final model evaluation.

PCA reduces the pre-processed spectral data to a small number of principal components (PCs) responsible for the majority of the spectral data variance [9]. Each PC is orthogonal to each other and they are generated in a decreasing order of explained variance, where the first PC covers most of the data variance, followed by the second PC and so on. Each PC is composed of scores and loadings, where the scores represent the variance on sample direction, hence being used to identify similarities/dissimilarities between samples; and the loadings represent the variance on wavenumber direction, being used to identify possible spectral biomarkers responsible for class differentiation. In PCA-LDA, a LDA classifier is employed in the PCA scores space in order to systematically distinguish the samples using a Mahalanobis distance calculation [10]. PCA-LDA models were optimised using cross-validation venetian blinds with ten data splits.

The PCA-LDA models output in the validation set (blind spectra) are used to calculate quality metrics or figures of merit in order to evaluate the model classification performance. Metrics such as accuracy (total number of samples correctly classified considering true and false negatives), sensitivity (proportion of positive observations correctly classified) and specificity (proportion of negative observations correctly classified) are calculated as follows [11]:

1 $$ \mathrm{Accuracy}\ \left(\%\right)=\left(\frac{\mathrm{TP}+\mathrm{TN}}{\mathrm{TP}+\mathrm{FP}+\mathrm{TN}+\mathrm{FN}}\right)\times 100 $$

2 $$ \mathrm{Sensitivity}\ \left(\%\right)=\left(\frac{\mathrm{TP}}{\mathrm{TP}+\mathrm{FN}}\right)\times 100 $$

3 $$ \mathrm{Specificity}\ \left(\%\right)=\left(\frac{\mathrm{TN}}{\mathrm{TN}+\mathrm{FP}}\right)\times 100 $$

where TP stands for true positives, TN for true negatives, FP for false positives and FN for false negatives.

## Results

### Case 1 (de novo OAC)

**Fig. 2:**
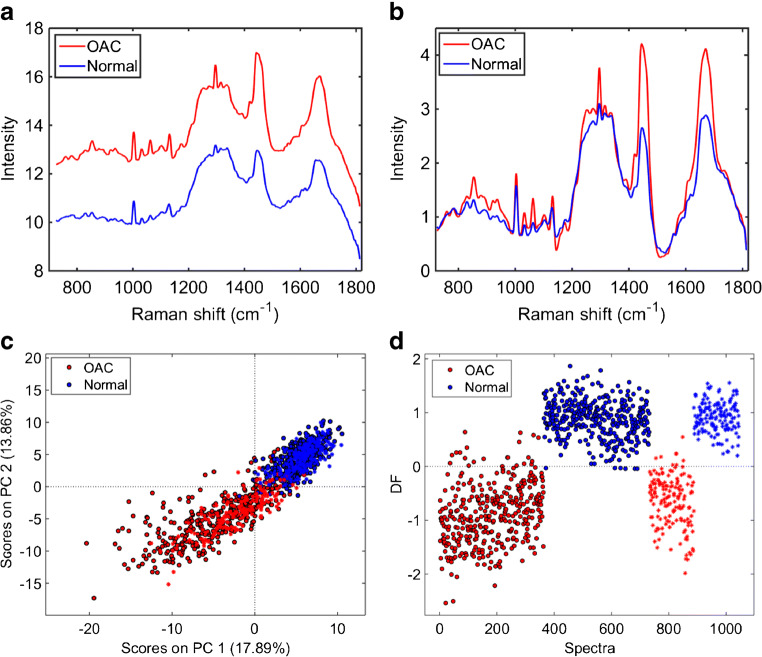
**a** Average raw Raman spectrum for OAC and normal tissue (case 1). **b** Average pre-processed (Savitzky–Golay smoothing [21 points window, 2nd order polynomial fitting] and AWLS baseline correction) Raman spectrum for OAC and normal tissue (case 1). **c** PCA scores plot for OAC and normal tissue. **d** PCA-LDA discriminant function (DF) plot for OAC and normal tissue (case 1)

The average raw and pre-processed (Savitzky–Golay smoothing and AWLS baseline correction) Raman spectra for normal and OAC tissue are depicted in Fig. 2 a and b, respectively. In Fig. 2b, there are clear spectral differences between normal and OAC tissue, especially in the regions between 800–1000 and 1240–1280 cm^−1^, and at the peaks at 1296, 1442 and 1670 cm^−1^, where OAC has a higher intensity than the normal tissue. The PCA scores plot (Fig. 2c) shows a clear natural difference between OAC and normal tissue along both PC1 (17.89% explained variance) and PC2 (13.86% explained variance). A supervised classification via PCA-LDA using 5 PCs (37% explained variance) shows a very clear separation between the two tissue types (Fig. 2d), where most of the spectra in the training and validation sets are correctly classified with an accuracy of 97% (94% sensitivity and 100% specificity) in validation (see Electronic supplementary material (ESM) Table S1).

**Fig. 3:**
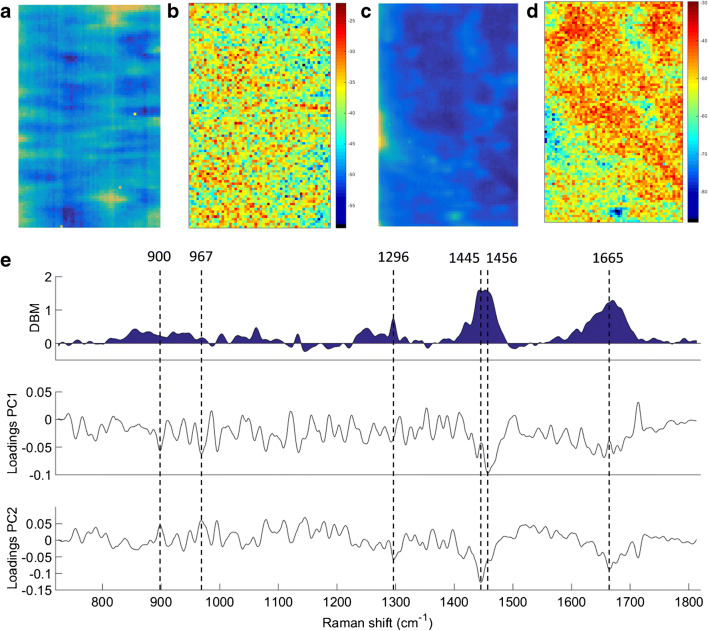
**a** Raw and **b** PCA-recovered images for normal tissue. **c** Raw and **d** PCA-recovered images for OAC tissue. **e** Difference-between-mean (DBM) spectrum and PCA loadings between normal tissue vs. OAC (case 1). Colour bar: mean relative intensity

The raw and reconstructed Raman mapping after PCA for normal tissue and OAC are shown in Fig. 3a–d, and the difference-between-mean (DBM) spectrum for normal vs. OAC tissue along with the PCA loadings on PC1 and PC2 are shown in Fig. 3e. The reconstructed mapping after PCA clearly shows the areas with cancerous tissue in red. Six spectral markers were found as the most important discriminant features between normal tissue and OAC: 900 cm^−1^ (C–O–C skeletal mode in monosaccharides (β-glucose)), 967 cm^−1^ (lipids), 1296 cm^−1^ [phosphodioxy (PO_2_^−^)], 1445 cm^−1^ (CH_2_/CH_3_ angular deformation in collagen), 1456 cm^−1^ (CH_2_ deoxyribose) and 1665 cm^−1^ (amide I of collagen) [12] (Fig. 3e). Peaks at around 900, 1440 and 1660 cm^−1^ are indicators of cancerous tissue, as well as changes in lipids, collagen and amide I peaks [13]. Changes in deoxyribose-phosphate spectral signatures have been also detected in cancer cells, which suggest partial destruction of the phosphate backbone [13].

### Case 2 (Barrett’s oesophagus to OAC)

**Fig. 4:**
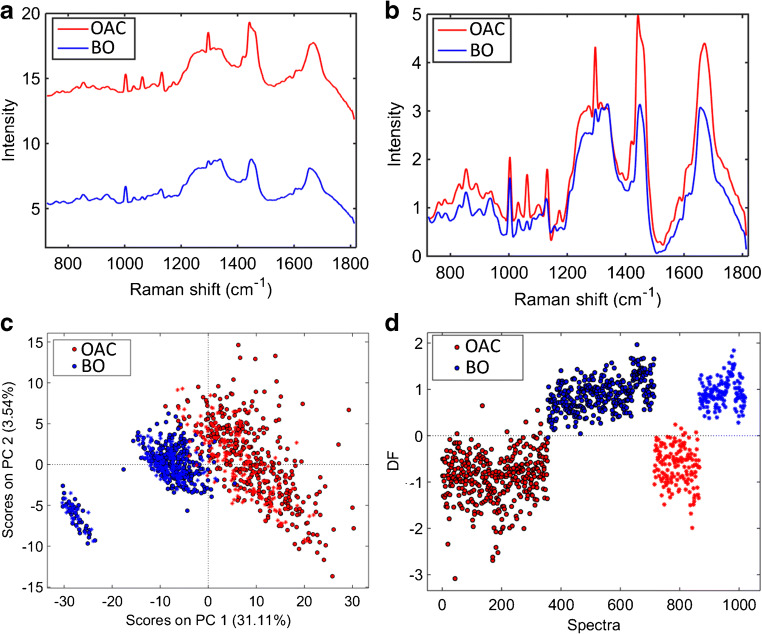
**a** Average raw Raman spectrum for OAC and Barrett’s oesophagus tissue (case 2). **b** Average pre-processed (Savitzky–Golay smoothing [21 points window, 2nd order polynomial fitting] and AWLS baseline correction) Raman spectrum for OAC and Barrett’s oesophagus tissue (case 2). **c** PCA scores plot for Barrett’s oesophagus tissue and OAC tissue. **d** PCA-LDA discriminant function (DF) plot for OAC and Barrett’s oesophagus tissue (case 2)

Figure 4a and b show, respectively, the average raw and pre-processed (Savitzky–Golay smoothing and AWLS baseline correction) Raman spectra for Barrett’s oesophagus and OAC tissue. In Fig. 4b, there are clear spectral differences between Barrett’s oesophagus and OAC tissue, where OAC tissue has an overall higher Raman intensity than Barrett’s oesophagus tissue through the whole spectrum. The PCA scores plot (Fig. 4c) shows a clear natural difference between Barrett’s oesophagus and OAC tissue especially along PC1 (31.11% explained variance). PCA-LDA using 3 PCs (38% explained variance) shows a clear separation between the two tissue types (Fig. 4d), where only a few OAC spectra are inside the Barrett’s oesophagus class space. This PCA-LDA model generated an accuracy of 98% (97% sensitivity and 100% specificity) to distinguish Barrett’s oesophagus tissue vs. OAC (ESM Table S2). OAC in this case largely originates from the Barrett’s oesophagus mucosa.

**Fig. 5:**
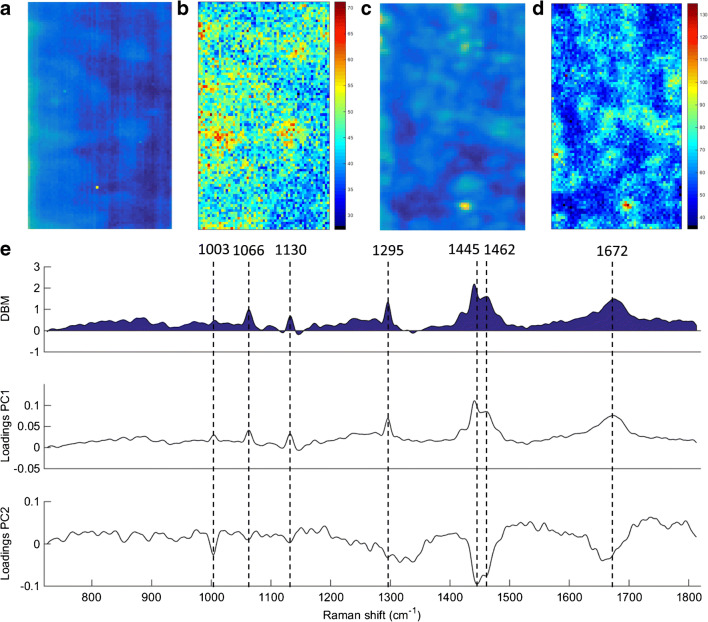
**a** Raw and **b** PCA-recovered images for Barrett’s oesophagus tissue. **c** Raw and **d** PCA-recovered images for OAC tissue. **e** Difference-between-mean (DBM) spectrum and PCA loadings between OAC vs. Barrett’s oesophagus tissue (case 2). Colour bar: mean relative intensity

The raw and reconstructed Raman mapping after PCA for Barrett’s oesophagus tissue and OAC tissue are shown in Fig. 5a–d, and the DBM spectrum for Barrett’s oesophagus tissue vs. OAC tissue along with the PCA loadings on PC1 and PC2 are shown in Fig. 5e. The reconstructed mapping after PCA shows the areas with OAC tissue in yellowish/light blue colour and the Barrett’s oesophagus tissue areas in yellow/red colour. Seven spectral markers were found as the most important discriminant features between OAC and Barrett’s oesophagus tissue: 1003 cm^−1^ (C–C skeletal in phenylalanine), 1066 cm^−1^ (proline/collagen), 1130 cm^−1^ (phospholipid structural changes (*trans* vs. *gauche* isomerism)), 1295 cm^−1^ (CH_2_ angular deformation), 1445 cm^−1^ (CH_2_/CH_3_ angular deformation in collagen), 1462 cm^−1^ (CH_2_ angular deformation in disaccharides) and 1672 cm^−1^ (amide I (C=O stretching coupled to a N–H bending)) [12] (Fig. 5e).

### Case 3 (Barrett’s oesophagus to OAC)

**Fig. 6:**
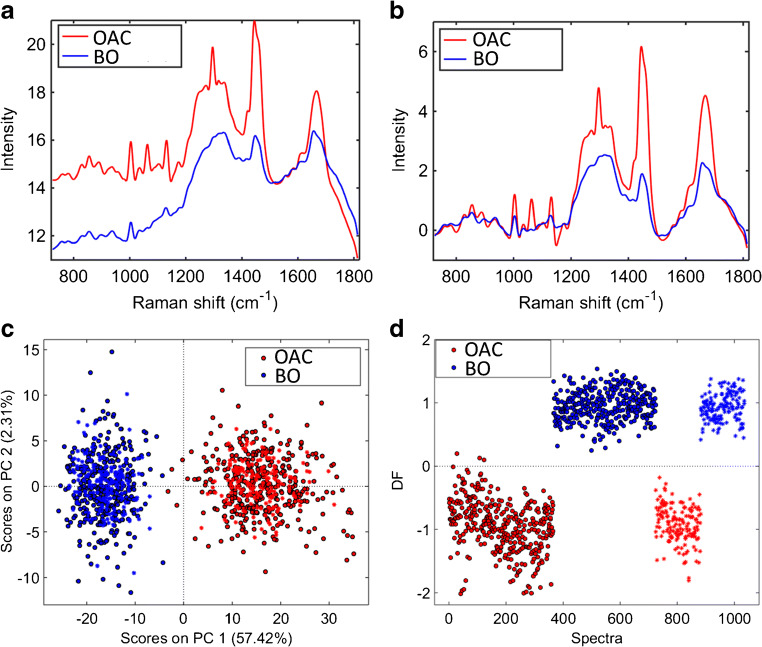
**a** Average raw Raman spectrum for OAC and Barrett’s oesophagus tissue (case 3). **b** Average pre-processed (Savitzky–Golay smoothing [21 points window, 2nd order polynomial fitting] and AWLS baseline correction) Raman spectrum for OAC and Barrett’s oesophagus tissue (case 3). **c** PCA scores plot for Barrett’s oesophagus and OAC tissue (case 3). **d** PCA-LDA discriminant function (DF) plot for OAC and Barrett’s oesophagus tissue (case 3)

The average raw and pre-processed (Savitzky–Golay smoothing and AWLS baseline correction) Raman spectra for the second case of Barrett’s oesophagus and OAC tissue are depicted in Fig. 6a and b, respectively. In Fig. 6b, there are clear spectral differences between OAC and Barrett’s oesophagus tissue, where OAC has higher Raman intensity especially in the regions between 1200–1500 and 1600–1700 cm^−1^. The PCA scores plot (Fig. 6c) shows a clear natural difference between OAC and Barrett’s oesophagus tissue along PC1 (57.42% explained variance). A supervised classification via PCA-LDA using 2 PCs (60% explained variance) shows an almost perfect separation between the two tissue types (Fig. 6d), where the spectra in the validation set were correctly classified with an accuracy of 100% (100% sensitivity and specificity) (ESM Table S3).

**Fig. 7:**
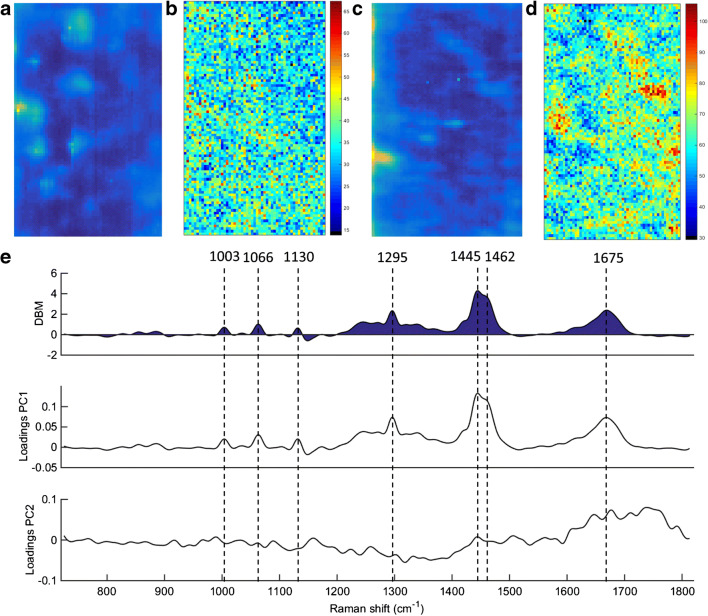
**a** Raw and **b** PCA-recovered images for Barrett’s oesophagus tissue. **c** Raw and **d** PCA-recovered images for OAC tissue. **e** Difference-between-mean (DBM) spectrum and PCA loadings between Barrett’s oesophagus vs. OAC tissue (case 3). Colour bar: mean relative intensity

Figure 7a–d show the raw and reconstructed Raman mapping after PCA for Barrett’s oesophagus and OAC tissue (case 3), and the DBM spectrum and PCA loadings on PC1 and PC2 for Barrett’s oesophagus vs. OAC tissue (case 3) are shown in Fig. 7e. The reconstructed mapping after PCA shows the areas with OAC tissue in a higher intensity yellow/red colour and the Barrett’s oesophagus tissue mapping in lower intensity yellow. Seven spectral markers were found as the most important discriminant features between OAC and Barrett’s oesophagus tissue (case 3): 1003 cm^−1^ (C–C skeletal in phenylalanine), 1066 cm^−1^ (proline/collagen), 1130 cm^−1^ (phospholipid structural changes (*trans* vs. *gauche* isomerism)), 1295 cm^−1^ (CH_2_ angular deformation), 1445 cm^−1^ (CH_2_/CH_3_ angular deformation in collagen), 1462 cm^−1^ (CH_2_ angular deformation in disaccharides) and 1675 cm^−1^ (amide I) [12]. The same spectral markers observed in case 2 (Barrett’s oesophagus vs. OAC) were found in case 3 (Barrett’s oesophagus vs. OAC), confirming the consistency of this spectral methodology to provide repetitive results in different patients and that these seven spectral markers are highly associated with a chemical difference between Barrett’s oesophagus and OAC tissue.

## Discussion

Raman spectra can be extrapolated as a direct function of the molecular composition of tissue. Thus, there is potential that Raman can be utilised as a pathological tool in validating diagnoses. Raman spectroscopy has been utilised with promising results in the fields of neurosurgery [14, 15] and gynaecology [16, 17] using both biofluids and human tissue.

Morphological classification of certain tumours is becoming more difficult even with the advent of staining and other histopathological adjuncts. With such variability in malignant disease as well as the need for more accurate classification systems, spectroscopy has expanded as an aid in tissue diagnosis. Using sophisticated calibration transfer procedures, different sources of variation can be normalised into a single model using computational-based methods. This can generate measurements performed under different conditions which generate the same result, eliminating the need for a full recalibration [18]. This could prove to be an invaluable tool when evaluating data from different laboratories throughout the UK.

Better understanding of the carcinogenesis of Barrett’s oesophagus is an essential step in targeting the disease and improving survival. The potential of the present endoscopic surveillance programmes to improve detection of adenocarcinoma at an early stage has been questioned by many studies [19, 20]. Case 1 is highly unusual as finding OAC in a patient after a normal OGD 3 months prior is rare. Furthermore, only a minority of patients progress from metaplasia to low- and high-grade dysplasia (0.12–0.6% annually) [21, 22]. Cases 2 and 3 describe patients with an OAC diagnosis only 5 years post initial Barrett’s oesophagus diagnosis. Few studies have directly analysed spectral mapping in the same index patient, particularly in patients where the timing of diagnosis between benign disease and malignancy is ≤ 3 months. In fact, we are of the opinion that this is quite unique.

There have been numerous applications of Raman spectroscopy for quantitative ex vivo sample analysis [23–27]. These studies all range from diagnostic accuracies for establishing non-dysplastic to dysplastic tissue from 88 to 97% [23–27]. This compares with our group who has established an accuracy of 97% (94% sensitivity and 100% specificity) between normal squamous epithelium and OAC. Furthermore, our group has demonstrated a 100% accuracy (98% sensitivity; 100% specificity) between Barrett’s oesophagus and OAC.

The next stages of analysis would be in an in vivo setting. The largest study to date interrogating Barrett’s oesophagus specimens was performed by Bergholt et al. [28] where 373 patients subjected to multimodal real-time optical imaging were included. The authors focused on three groups (columnar-lined oesophagus without goblet cells, *n* = 907 spectra; non-dysplastic Barrett’s oesophagus, *n* = 318 spectra; Barrett’s oesophagus positive for HGD, *n* = 177 spectra). Their method generated 79% sensitivity and 74% specificity for detection of OAC.

Bergholt et al. [29] performed a smaller in vivo study where a total of 75 oesophageal tissue sites from 27 patients were measured. An optical probe comprising a central fibre of 200 μm for delivery of the laser light (785 nm wavelength) to the tissue surrounded by thirty-two 200 μm collection fibres was introduced to establish real-time spectra. Forty-two in vivo Raman spectra were acquired on normal tissues and 33 on malignant tumours (adenocarcinoma, *n* = 27; squamous cell carcinoma, *n* = 6) as confirmed by histopathology. The OAC tissue showed distinct Raman signals associated with cell proliferation, lipid reduction, abnormal nuclear activity and neovascularisation. Using a linear discriminant analysis algorithm, the authors demonstrated an accuracy of 96% (sensitivity of 97.0% and specificity of 95.2%) for in vivo diagnosis of oesophageal malignancy.

Multiple studies have also qualified that the concentration of particular biomolecules elicited from Raman spectroscopy including phospholipids, proteins and collagen increases from normal squamous epithelial tissue to dysplastic tissue [24–27, 30, 31]. This is in keeping with our findings as these cases have clearly demonstrated spectral markers mainly β-glucose, lipids, phosphodioxy group, deoxyribose and collagen changes associated with differences between normal squamous epithelium and OAC tissue, and phenylalanine, proline/collagen, phospholipids, disaccharides and protein peaks associated with differences between Barrett’s and OAC tissue. The findings are particularly interesting as mapping analysis was performed directly comparing tissue in the same index patients. The subtle spectral differences established in the normal squamous epithelial tissue sample may suggest its propensity in developing OAC later down the line.

## Conclusion

Establishing dichotomous biomarkers of commitment in tissue at a non-dysplastic stage [32] can give clues to the propensity of developing OAC. Finding these markers early would prevent costly further invasive management requiring extensive treatment including chemotherapy, chemoradiotherapy and/or surgical resection. This reinforces the potential of using Raman microspectroscopy in clinical translation, where sample diagnosis can be obtained in a computer-automated, minimally destructive, fast and accurate manner. These preliminary results need further substantive prospective studies in an in vivo setting to confirm the results and to study more biochemical components, which may be elevated with the level of dysplasia encountered.

## A.Electronic supplementary material

ESM 1 (PDF 89.4 kb).
