# Supplementary material for: Establishing spectrochemical changes in the natural history of oesophageal adenocarcinoma from tissue Raman mapping analysis
Source: Anal Bioanal Chem. 2020 Apr 25;412(17):4077–87. doi: 10.1007/s00216-020-02637-1 (PMC7320044; doi:10.1007/s00216-020-02637-1)
Supplement: Supplementary file 1 — (PDF 89.4 kb). [file 216_2020_2637_MOESM1_ESM.pdf]

Analytical and Bioanalytical Chemistry

Electronic Supplementary Material

Establishing spectrochemical changes in the natural history of oesophageal adenocarcinoma from tissue Raman mapping analysis

Ishaan Maitra, Camilo L. M. Morais, Kássio M. G. Lima, Katherine M. Ashton,
Danielle Bury, Ravindra S. Date, Francis L. Martin

Table S1 Quality parameters (accuracy, sensitivity and specificity) for distinguishing OAC *vs.* Normal (case 1) tissue using PCA-LDA

Dataset	Accuracy	Sensitivity	Specificity
Training	96%	94%	98%
Cross-validation	96%	94%	99%
Validation	97%	94%	100%

Table S2 Quality parameters (accuracy, sensitivity and specificity) for distinguishing OAC *vs.* BO (case 2) tissue using PCA-LDA

Dataset	Accuracy	Sensitivity	Specificity
Training	99%	98%	100%
Cross-validation	99%	98%	100%
Validation	98%	97%	100%

Table S3 Quality parameters (accuracy, sensitivity and specificity) for distinguishing OAC *vs.* BO (case 3) tissue using PCA-LDA

Dataset	Accuracy	Sensitivity	Specificity
Training	100%	99%	100%
Cross-validation	100%	99%	100%
Validation	100%	100%	100%
